# Biodegradable Cable-Tie Rapamycin-eluting Stents

**DOI:** 10.1038/s41598-017-00131-w

**Published:** 2017-03-08

**Authors:** Cheng-Hung Lee, Ming-Jer Hsieh, Shang-Hung Chang, Chang-Lin Chiang, Ching-Lung Fan, Shih-Jung Liu, Wei-Jan Chen, Chao-Jan Wang, Ming-Yi Hsu, Kuo-Chun Hung, Chung-Chuan Chou, Po-Cheng Chang

**Affiliations:** 1 Division of Cardiology, Department of Internal Medicine, Chang Gung Memorial Hospital-Linkou, Chang Gung University College of Medicine, Taipei, Taiwan; 2 Department of Mechanical Engineering, Chang Gung University, Taoyuan, Taiwan; 3 Department of Orthopedic Surgery, Chang Gung Memorial Hospital-Linkou, Taoyuan, Taiwan; 4 Department of Medical Imaging and Intervention, Chang Gung Memorial Hospital-Linkou, Taoyuan, Taiwan

## Abstract

“Cable-tie” type biodegradable stents with drug-eluting nanofiber were developed to treat rabbit denuded arteries in this study. Biodegradable stents were fabricated using poly-L-lactide film following being cut and rolled into a cable-tie type stent. Additionally, drug-eluting biodegradable nanofiber tubes were electrospun from a solution containing poly (lactic-co-glycolic acid), rapamycin, and hexafluoroisopropanol, and then mounted onto the stents. The fabricated rapamycin-eluting cable-tie stents exhibited excellent mechanical properties on evaluation of compression test and collapse pressure, and less than 8% weight loss following being immersed in phosphate-buffered saline for 16 weeks. Furthermore, the biodegradable stents delivered high rapamycin concentrations for over 4 weeks and achieved substantial reductions in intimal hyperplasia associated with elevated heme oxygenase-1 and calponin level on the denuded rabbit arteries during 6 months of follow-up. The drug-eluting cable-tie type stents developed in this study might have high potential impacts for the local drug delivery to treat various vascular diseases.

## Introduction

Percutaneous angioplasty with metallic stents has been used for decades to open up blocked arteries and restore blood flow to the distal circulation that is thought to be causing myocardial or limb ischemia^1, 2^. However, bare metal stents (BMS) have been largely replaced for coronary artery disease (CAD) because drug-eluting stents (DES) have substantially decreased the incidence for repeat revascularization^3^. Nevertheless, concerns have been raised about delayed endothelinization in DES resulted in the risk of late stent thrombosis^4, 5^. Until healing of the vessel occurs through re-endothelialization, the use of stenting should be non-permanent and limited to following shortly thereafter by the intervention, and responses to the durable polymer could be a problem for chronic inflammation and late stent thrombosis^6–8^. Hence, subsequent generations of DES have concentrated on biodegradable drug-eluting polymers and the development of biodegradable scaffold-based stents.

Since the first biodegradable stent^9^ in the 1990s, attempts have been made to develop such stents for cardiovascular applications^10–12^. However, the expansion of most of these stents relies heavily upon the elastic recovery behavior of the polymeric scaffolds. Even though the currently available biodegradable stents have shown relevant products, but still need an advanced manufacturing process^13^ or complex structure which may have stent fracture risk from degradation. Long-term restenosis might happen because of progressive recoil of the polymer-based stents with unsatisfactory elasticity or loss of elastic memory. Recently, a polylactide biodegradable platform eluting drug to treat CAD has desired outcomes comparable with metallic DES^14–16^. However, clinical trials show that those with a biodegradable vascular scaffold have a greater incidence of incomplete strut apposition and tissue prolapse area. Edge dissection and stent strut fracture may occur, leading to acute stent thrombosis and even death. Furthermore, a direct correlation with stent thrombosis has been observed with increasing extensive procedures, costing on average USD 11,134 per patient^17–19^.

Hybrid biocompatible nanofiber drug-eluting polymers on BMS and biodegradable-polymer stents have been developed for the application of drugs to repair denuded arteries^20–22^. In this study, a cable-tie poly-L-lactide (PLLA) stent loaded with rapamycin-eluting poly (lactic-co-glycolic acid) (PLGA) nanofibers was developed to treat denuded rabbit arteries.

## Materials and Methods

### Materials

The biodegradable polymers used in this study were PLLA (Resomer L209S), and PLGA with a poly (lactic acid): poly (glycolic acid) ratio of 75:25 (Resomer RG756S, Boehringer, Germany). We used the anti-proliferative drug rapamycin, and the solvents included chloroform and hexafluoroisopropanol (HFIP) (Sigma-Aldrich, MO, USA).

### Biodegradable drug-eluting stents

**Figure 1:**
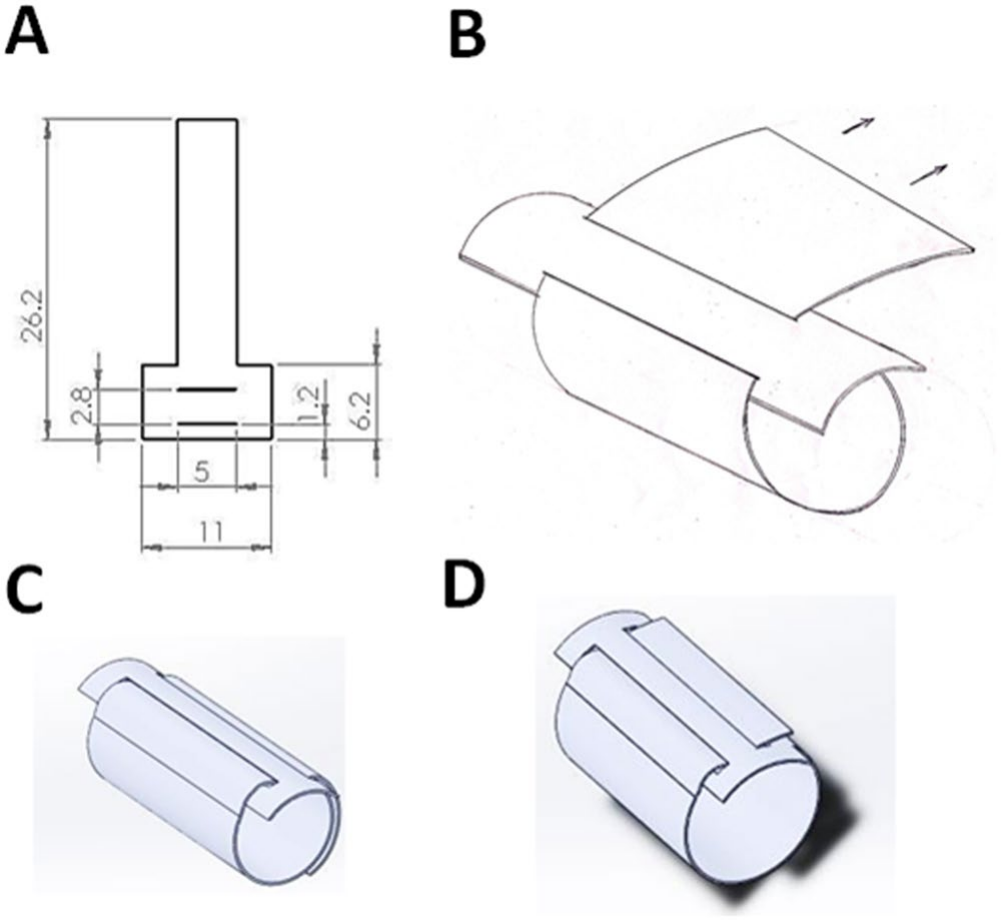
Dimensions of the stent components and the schematic design for self-lock characteristics. The stent element was cut from the film as shown (**A**). One side of the element (the strip) was glided through the two slits and rolled into a cable-tie type stent as shown (**B**). Before inflation, the stent can be tied on the preferred balloon size (**C**). Due to the geometry constrains of the double slits, once the stent is expanded, the strip is not able to slide backward (**D**).

PLLA film with thickness 60 μm was first prepared using a solvent-casting method with chloroform as the solvent. The stent element was cut from the film as shown in Fig. 1A. One side of the element (the strip) was passed through two slits and rolled into a cable-tie type stent as shown in Fig. 1B. The stent was expanded after being inflated by the balloon (Fig. 1C). Because of geometric constraints of the double slits, the strip could not slide backwards after stent expansion (Fig. 1D). The proposed stent thus exhibited a self-locking characteristic with negligible recoil when subjected to an external force.

**Figure 2:**
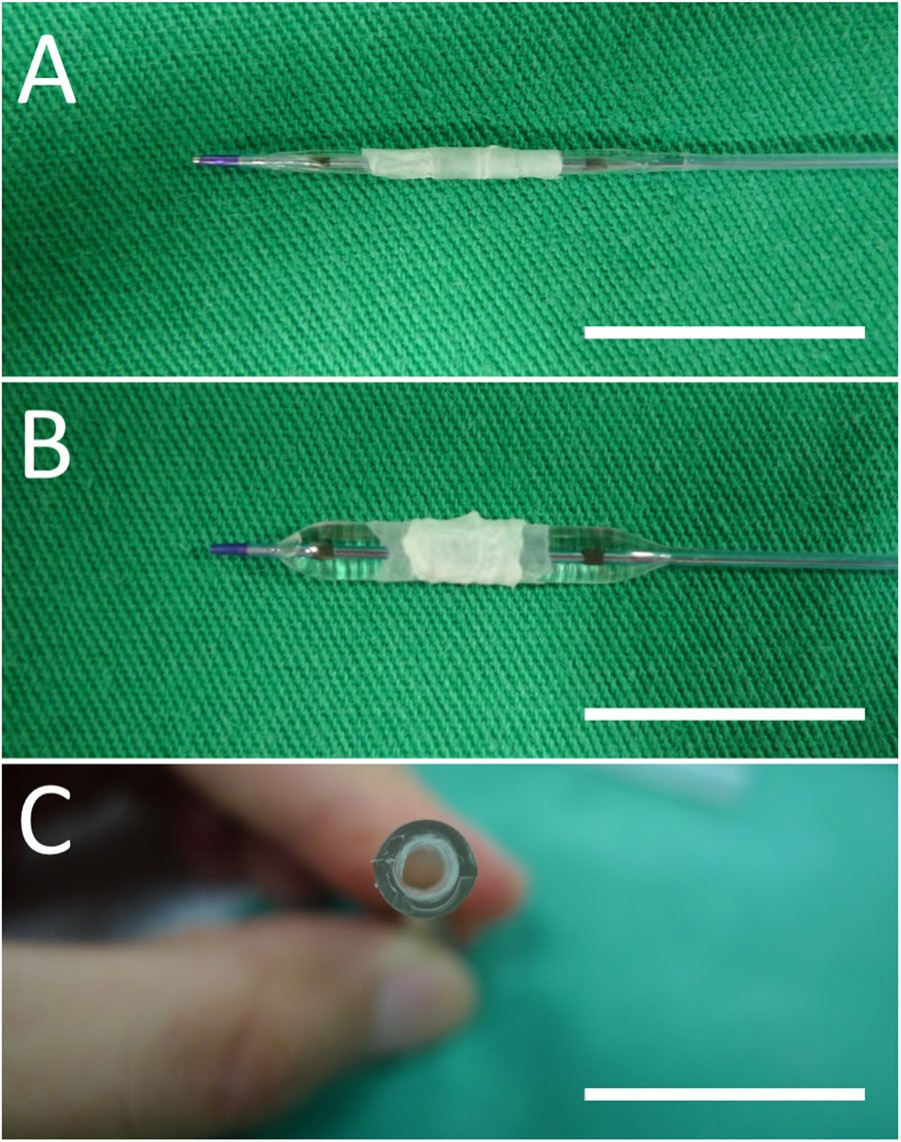
The biodegradable stents (**A**) before and (**B**) after expansion by a balloon (**C**) expansion on plastic tube (Scale bar = 15 mm).

Biodegradable nanofibers were prepared using an electrospinning technique. All electrospinning experiments were carried out at room temperature. PLGA (240 mg) and rapamycin (40 mg) were first dissolved in 1 ml of HFIP to electrospin nanofiberous membrane tubes. After electrospinning, the tubes of electrospun nanofibers were then hand crimped and mounted onto the PLLA stents (Fig. 2). All of the biodegradable drug-eluting stents were placed in a vacuum oven at 40 °C for three days to let the solvents evaporate.

Group A (n = 12) were biodegradable stents with rapamycin loading; and group B (n = 12) were stents with no drug loading; and group C (n = 6) were BMS (Gazelle, Bare Metal Coronary Stent, Biosensors Europe SA, Switzerland) with rapamycin loading.

### Compression test

Compression tests were conducted on a LLOYD tensiometer (AMETEK, USA). A 2500 N load cell was employed with a cross-head speed of 0.1 cm/min. In radial compression, the stent being tested as well as compressed to deform with different loads (30% reduction in the maximum deflection) was recorded. Five samples were tested for fabricated stents and commercial metallic stents (3.0 × 14 mm) (Gazelle, Bare Metal Coronary Stent, Biosensors Europe SA, Switzerland).

### Collapse pressure

A self-made system with a flexible Tygon tube attached to a pressure chamber and water circulating flow loop (37 °C; 10 L/min; 76 mmHg) was performed for the collapse pressure of the stents^13^ (n = 5).

### Weight retention of stent

Weight variation of the stent immersed in the 37 °C phosphate-buffered saline (PBS) was calculated. The test stents were taken away from the solution and weighed at different time points following being dried in an oven for one day (n = 5).

### Microscopic examination

The surface morphology of the electrospun nanofibers and the stents following various times of elution in the buffer solution was examined using scanning electron microscope (SEM) (Hitachi, Ltd, Tokyo, Japan). Diameters and pore space of 100 randomly selected fibers were measured and the average of these measurements gave the diameter and pore space of the nanofibers by ImageJ software (National Institutes of Health, Bethesda, MD, USA).

### *In-vitro* release of rapamycin

The glass test tubes with 3 ml of the PBS (0.15 mol/L, pH 7.4) at 37 °C were used for stent incubation and the dissolution sampling. The repeated procedure with adding new PBS (3 ml) for the every 24-hour period was carried out for 4 weeks.

### High-performance liquid chromatography (HPLC) study

The release of rapamycin for stents was evaluated using a Hitachi L2400 UV-VIS System (Hitachi, Ltd, Tokyo, Japan). The Hibar C-18, 5 μm, 4.6 × 250 mm HPLC column (Merck KGaA, Darmstadt, Germany) was performed for rapamycin separation. The mobile phase had acetonitrile, methanol, and distilled water (40/45/15, v/v/v) at a flow rate of 1.0 ml/min and dissolution sampling (n = 3) was monitored by absorbance at 278 nm.

### *In vivo* animal study

Thirty healthy, adult male New Zealand white rabbits (average weight: 3.4 ± 0.5 kg) were studied. All experimental procedures were approved by the Institutionally Animal Care and Use Committee of the Chang Gung University, and the aspects of rabbits housing were carried out under the supervision of a licensed veterinarian with the regulations of the Ministry of Health and Welfare, Taiwan.

With oxygen at 2 L/min through a face mask, Xylazine (9.3 mg/kg) (Bayer, Kiel, Gemerny) sedation and tiletamine-zolazepam (10 mg/kg) (VIRBAC, Carros, France) anesthesia were given by intramuscular injection. Group A (n = 12) received biodegradable stents with rapamycin loading; and group B (n = 12) received stents with no drug loading; and group C (n = 6) were treated with BMS with rapamycin loading. Endothelial denudation injury of the artery, and stents implantation procedures were performed as previous studies^20–22^.

Stented vessels were studied at 6 months after deployment for both inflammatory and injury reactions by histological examinations and microscopic observation^23, 24^. The scale of inflammation was 0 = none; 1 = mild, including minimal infiltrated inflammatory cells; 2 = moderate; 3 = severe with large clusters of inflammatory cells with granulomatous morphology. The scale of vessel injuries were: 0 = strut not in contact with internal elastic lamina (IEL); 1 = strut in contact with IEL and profile in neointima; 2 = strut penetrates IEL with profile in media; 3 = strut penetrates media and is in contact with external elastic lamina, and 4 = strut is in adventitia. The scores of all struts were averaged to obtain the mean score for each of the 24 histological sections.

The ImageJ software was also employed to evaluate the extent of the endothelial surface coverage above the stent struts^25^. The degrees of cell coverage on randomly selected areas were reported as endothelial coverage percentages. The iE33 ultrasound device (Philips Medical Systems, WA, USA) and angiography were used for hemocompatibility of the implanted stents and patency of the arteries. *In vivo* assessment of endothelial function by trans-abdominal ultrasound was carried out at 4 weeks^26^. Briefly, endothelium-dependent vasomotor function at the stented segments and at 20–25 mm from the non-stented and non-injured segments as a reference (control) was evaluated following the infusion of two incremental doses of acetylcholine (Sigma-Aldrich, MO, USA) (Ach, 0.05 and 0.5 μg/mL/min). Additionally, endothelium-independent function was analyzed using nitroglycerine (Nippon Kayaku, Tokyo, Japan) (5 μg/mL/min)^20^.

### Immunofluorescence and Western Blot Analysis

Optimal Cutting Temperature compound (OCT) (Tissue Tek, Tokyo, Japan) is used to embed tissue samples prior to frozen sectioning on a microtome-cryostat. Frozen sections were washed in PBS for 10 minutes and blocked with 2% bovine serum albumin (Sigma-Aldrich, MO, USA) for 40 minutes at room temperature. The sections were then incubated for one hour at room temperature with primary antibodies against heme oxygenase-1 (Ho-1), and calponin (Abcam, MA, USA, dilution 1:100), and secondary Cy3-conjugated antibody (Thermo Fisher Scientific, MA, USA, dilution 1:100). Nuclei were visualized by DAPI-staining.

Immunoblotting using anti-heme oxygenase-1 (HO-1), anticalponin (Dako), and antiGAPDH (Santa Cruz, Delaware Ave, CA) antibodies as primary antibodies was carried out. The amount of the proteins which were relative to GAPDH was analyzed using the enhanced chemiluminescence-detection method (Amersham, Netherlands) and quantified by densitometry.

### Statistics and data analysis

Statistical analysis was conducted with SPSS software (version 17.0 for Windows; SPSS Inc., Illinois, USA). All data are presented as means ± standard deviation. One-way ANOVA, followed by *post hoc* Bonferroni analysis for pairwise comparisons was used to compare the data for statistical significance. Differences were considered statistically significant at a *p* value of less than 0.05.

## Results

Using solvent-casting and electrospinning techniques, we successfully fabricated biodegradable drug-eluting stents. These biodegradable stents were passed over a commercial balloon dilatation catheter (Voyager NC Coronary Balloon Catheter 3.0 × 15 mm, Abbott Vascular, CA, USA) (Fig. 2A), and then placed inside a flexible plastic tube (3.0 mm) for inflation using a manual balloon catheter pump (Perouse Medical, France). The fabricated stents with the nanofibers were expanded with the force exertion by the balloon (8 atm) (Fig. 2B). The recoils of group A and B stents in the longitudinal dimension after expansion were comparable (diameter 3.09 ± 0.11 mm v.s. 3.04 ± 0.13 mm, *p* = 0.543). A “cable-tie” type stent with an internal diameter of 3.0 mm and an outer layer of rapamycin-eluting nanofibers was thus achieved. The stent still attached to the plastic tube following balloon pressure release and remove because of the anti-recoil property of the stent design (Fig. 2C).

**Figure 3:**
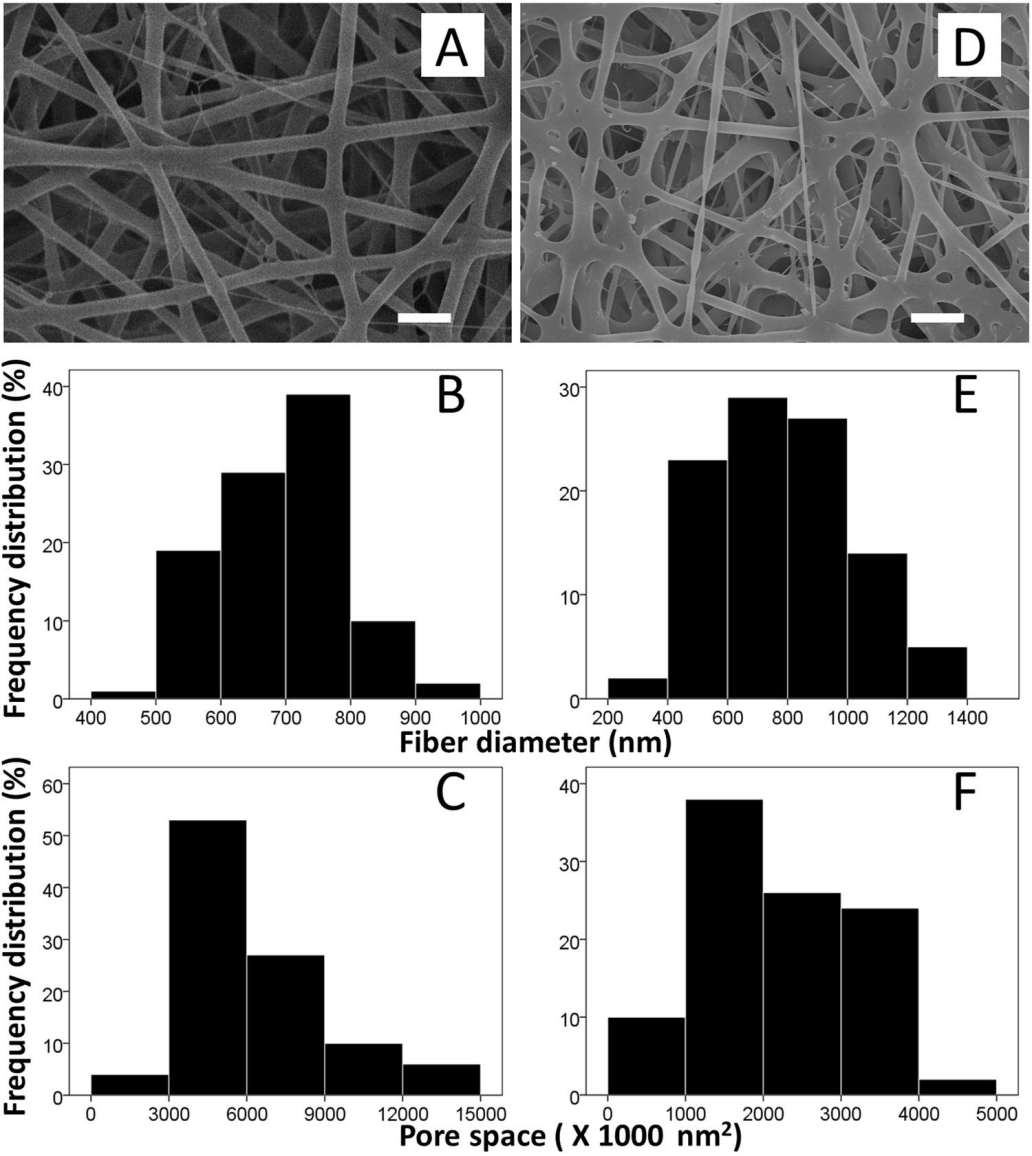
SEM view of electrospun drug-eluting nanofibers (n = 100 in each group) (**A**) rapamycin; and (**D**), PLGA only) and their frequency distribution of fiber diameter (**B** and **E**) as well as pore space (**C** and **F**) (Scale bar 2.5 *μ*m).

Figure 3 shows electrospun drug-eluting nanofibers (5,000X) using the SEM micrograph. The mean diameter of the nanofibers in group A (695.4 ± 98.8 nm) was significantly lower than that in group B (780.5 ± 226.9 nm) (*p* = 0.001) (Fig. 3B and E), however the pore space in group A (6701.2 ± 2683.7 × 10^3^ nm^2^) was significantly larger than that in group B (2196.4 ± 936.3 × 10^3^ nm^2^) (Fig. 3C and F) (p < 0.001).

### Characteristics of the fabricated stents

**Figure 4:**
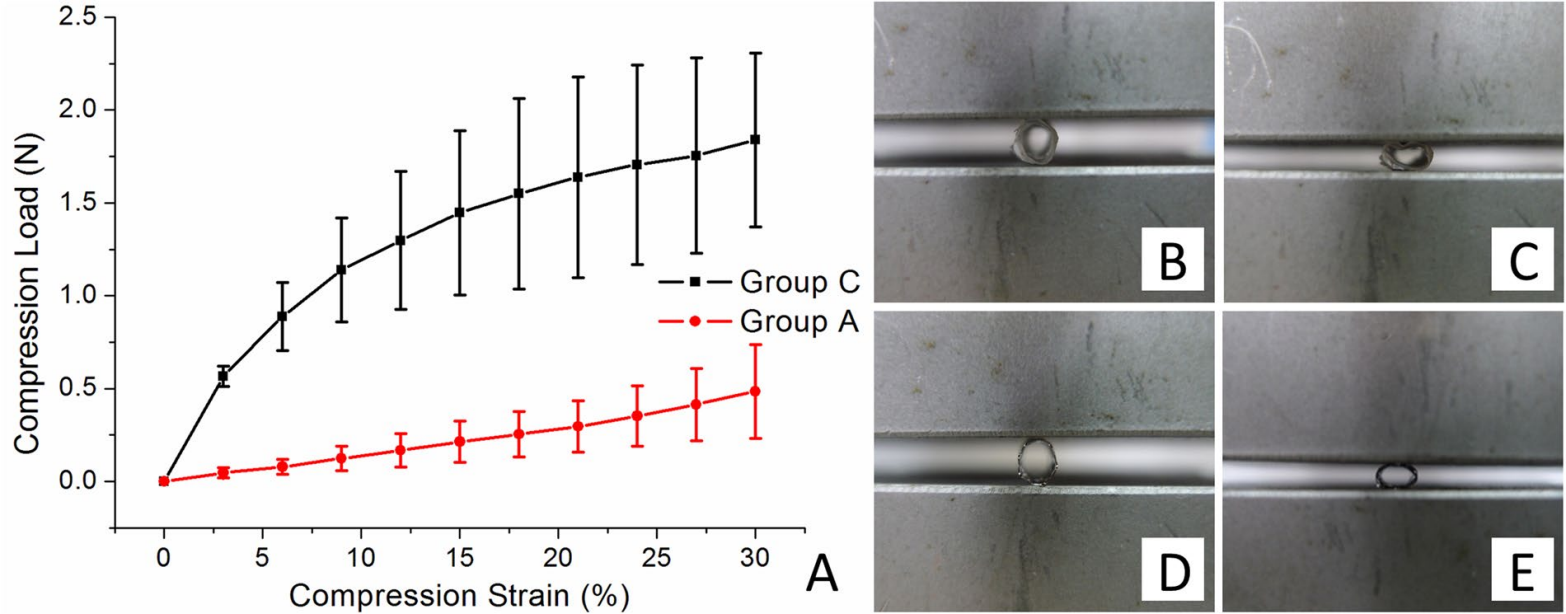
A comparison of the biodegradable stents (n = 5) and the metallic stents (n = 5) under the compression test (**A**) with maximum 30% compression strain (Initial diameter of the cable tie DES and BMS: (**B** and **D**) maximum 30% compression load: (**C** and **E**).

Tensile tester was used for compression strength of the fabricated stents to compare with that of metallic stents with maximum 30% compression strain (Fig. 4). As expected, the biodegradable stents had an inferior compression strength (0.48 ± 0.25 N) to the metallic stents (1.84 ± 0.47 N) on 30% load (*p* < 0.001) (initial diameter of the cable tie DES and BMS: B and D; maximum 30% compression load: C and E).

**Figure 5:**
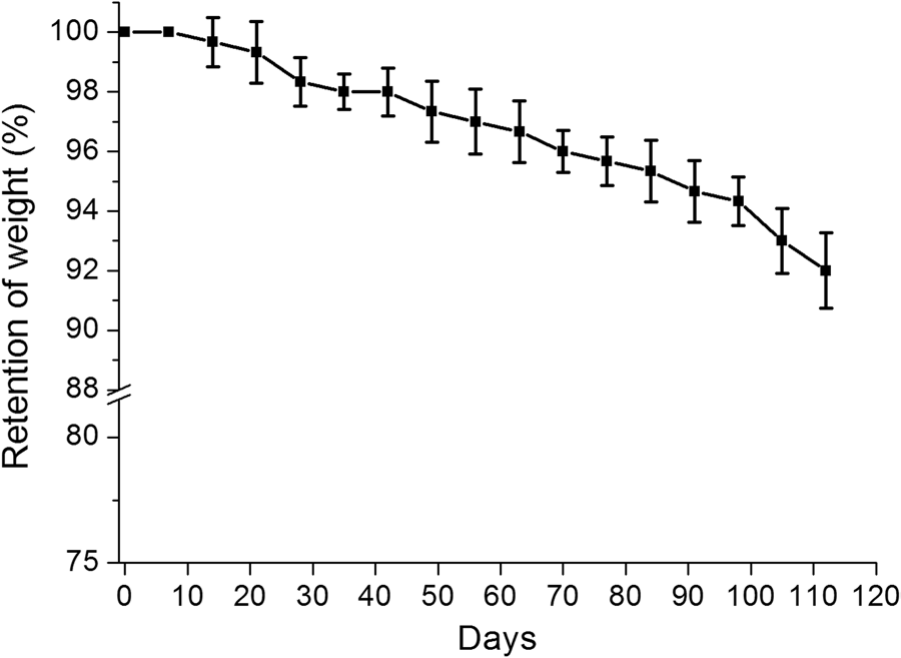
Weight change of the biodegradable stents with time (n = 5).

Both metallic and fabricated stents were also measured for collapse pressures. The collapse pressure of the metallic stent was 2.53 ± 0.11 kg/cm^2^, compared to 1.36 ± 0.14 kg/cm^2^ of the cable-tie biodegradable stent (*p* < 0.001). Figure 5 shows variations in weight of the fabricated stents with time. The weight of the biodedradable stents decreased to 92 ± 1.3% at 16 weeks, indicating that they underwent material degradation over time.

**Figure 6:**
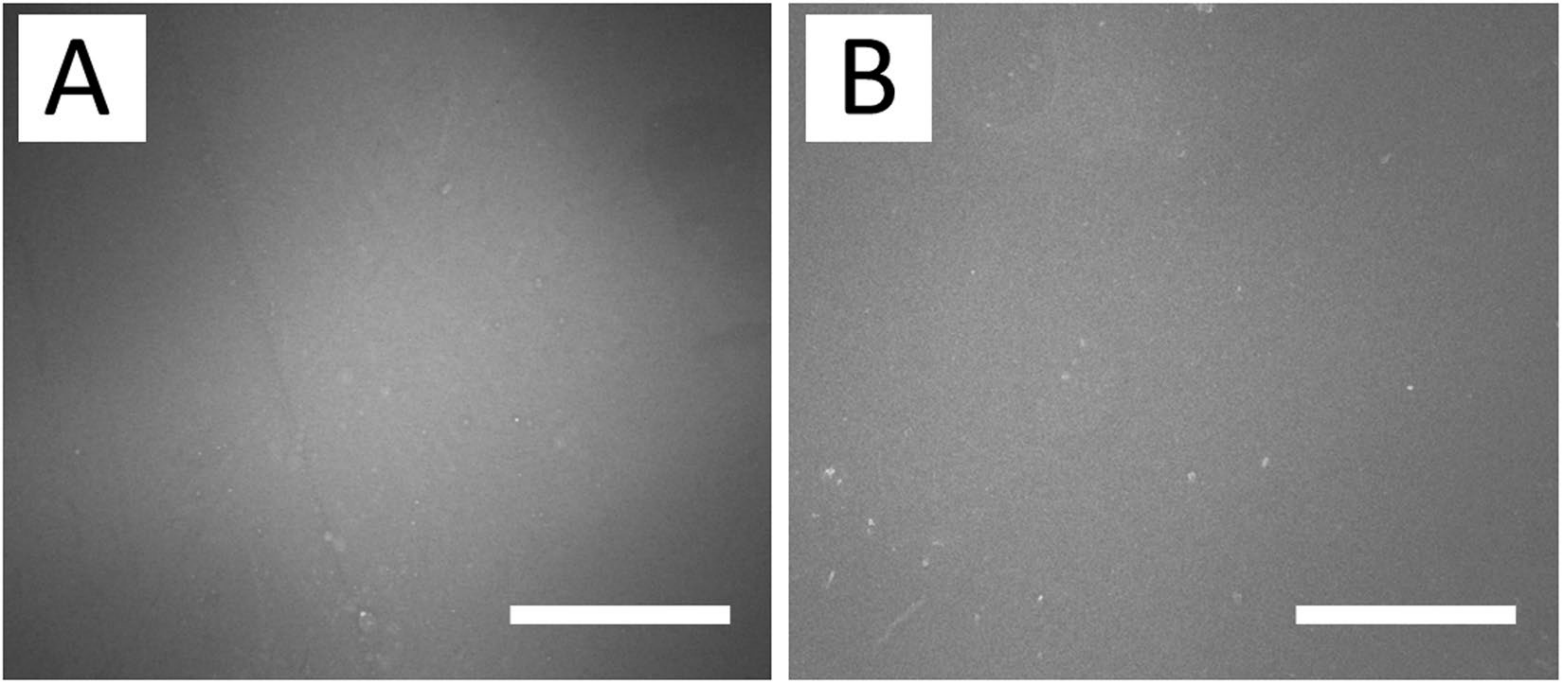
SEM photo of the stent surface, (**A**) before elution, and (**B**) 16 weeks after the elution process (n = 5) (scale bar = 50 μm).

The surface of a newly fabricated PLLA stent is presented using SEM in Fig. 6. The surface was smooth, despite some roughness that was due to the mold surface during the solvent casting process (Fig. 6A). Figure 6B shows micrographs of a fabricated stent after 16 weeks elution in PBS at 37 °C. On surface area, no significant appearances of stent degradation on surface were noted (500X) according to SEM.

### *In vitro* release of rapamycin

**Figure 7:**
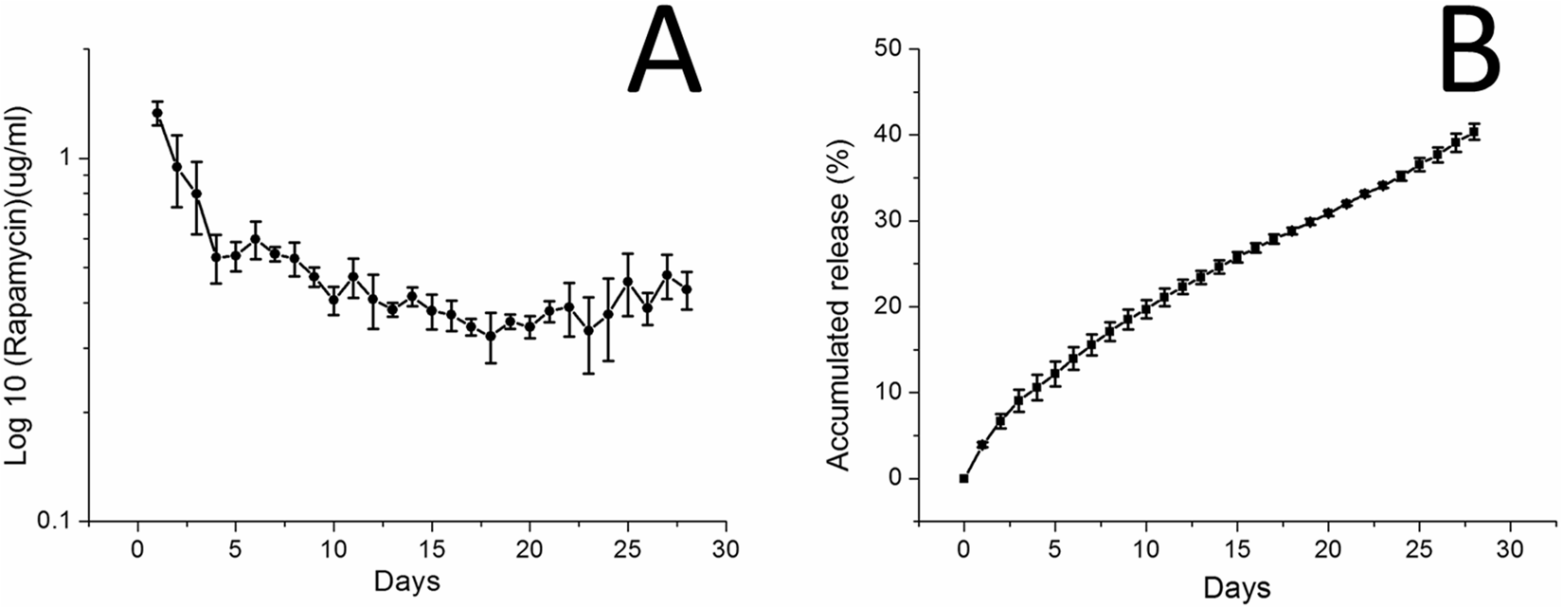
(**A**) Daily and (**B**) accumulated release curves of rapamycin from the biodegradable drug-eluting stents.

HPLC analysis suggested that the release curve of the biodegradable stents involved two stages of rapamycin release: an initial burst, and a rather steady degradation-controlled drug release (Fig. 7). Only 40% of the drugs were detected for the first 28 days, indicating that the drug-eluting nanofiber could effectively release rapamycin for more than four weeks.

### *In vivo* efficacy of the biodegradable stents

**Figure 8:**
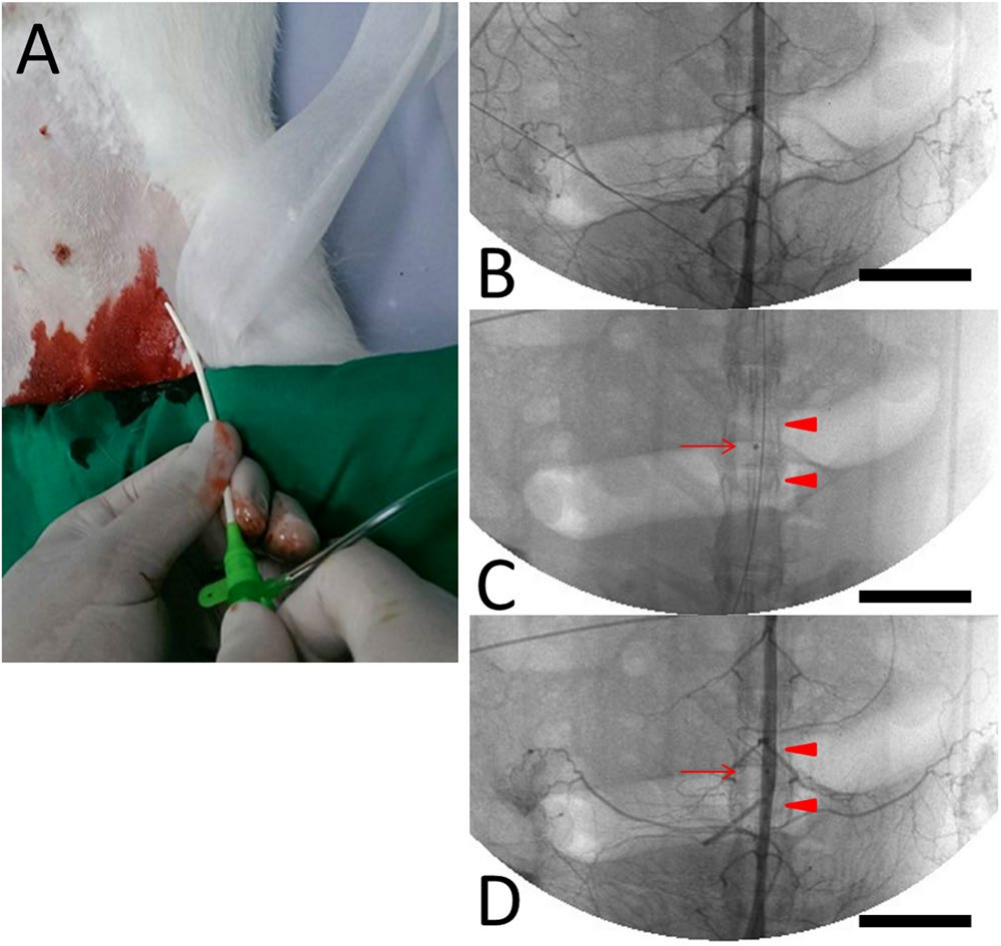
Using the puncture technique, a 6F sheath was inserted into the femoral artery (**A**). Biodegradable stents (arrowheads) with a single central radiopaque platinum marker (arrow), mounted and loaded with rapamycin nanofibers (6 μg/mm^2^) were implanted in the abdominal aorta of the rabbit (**B,C,D**) (Scale bar = 20 mm).

*In vivo* studies were evaluated using 30 rabbits that treated biodegradable cable-tie stents loaded with or without rapamycin or BMS with rapamycin in the denuded descending abdominal aorta. The vessel position and stent deployment were verified via angiography in each animal during the procedure. Since the stents themselves (arrowheads) could not be visualized under X-rays, we mounted a platinum marker bead (arrow) in the struts of the middle section (Fig. 8).

**Figure 9:**
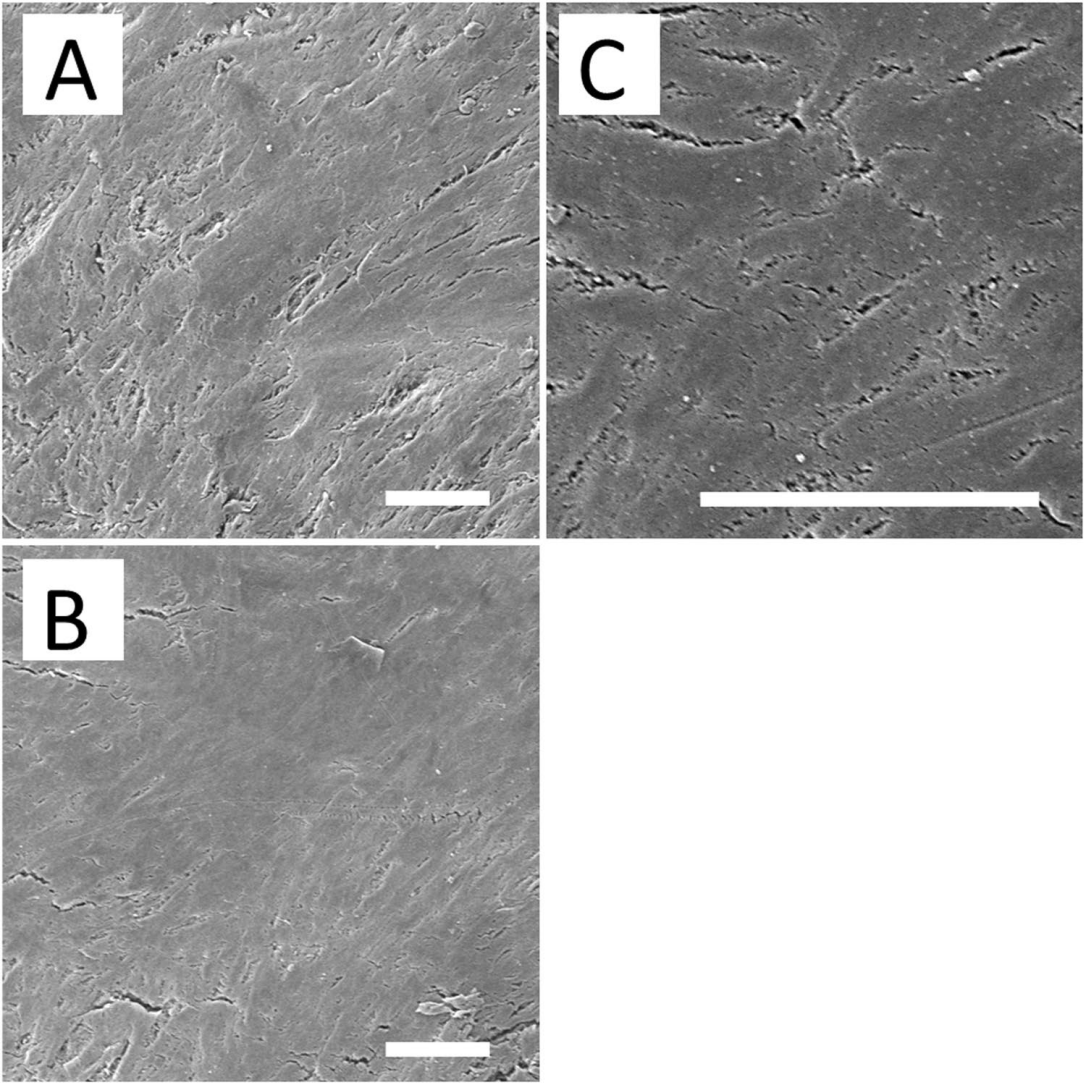
At 6 months, the surfaces of the struts were completely covered with regularly shaped endothelia in close contact with each other in Group A and B (300X) (**A** and **B**). Furthermore, re-endothelialization on the surfaces of struts among and the endothelia of group A were aligned in the direction of the flow (1000X) (**C**). (Scale bar = 50 *μ*m).

Figure 9 shows the observed endothelial coverage of the animals’ arteries. At 6 months, the surfaces of the struts were completely covered with regularly shaped endothelia in close contact with each other (300X) (Fig. 9A and B) (Endothelial coverage percentage: group A 97.1 ± 1.5, group B 97.3 ± 1.3, group C 96.2 ± 0.9, Anova *p* = 0.145) (Fig. 9A). Therefore, re-endothelialization on the surfaces of the struts and the endothelia of group A were aligned in the direction of the flow and comparable with those of group B with no drug loading (1000X) (Fig. 9B).

**Figure 10:**
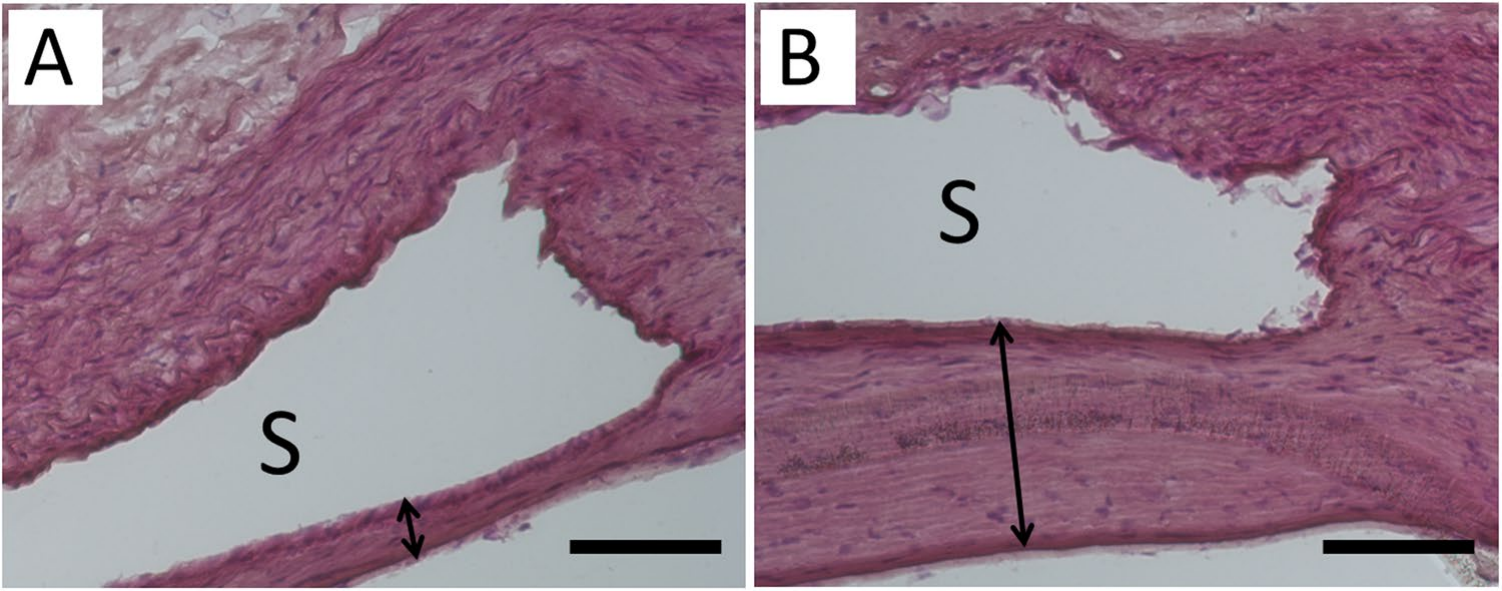
By weeks 24, group A exhibited re-constituted endothelium with insignificant intimal hyperplasia (**A**) however, group B showed substantial intimal hyperplasia with a thickness of around 70 μm (**B**) (double arrow). (Scale bar = 50 *μ*m) (S: stent strut).

Following incision of the aorta, the implanted stents were found to be intact. Additionally, no thrombus formation in the stent-implanted vessels was noted in the photomicrographs of cross-sections (Fig. 10). By week 24, group A exhibited re-constituted and regenerated endothelium without substantial intimal hyperplasia (Fig. 10A), whereas group B demonstrated significant intimal hyperplasia (Fig. 10B, double arrow). No significant variation for inflammatory response was detected among the three groups of rabbits (1.5 ± 0.5 vs. 1.4 ± 0.5 vs. 1.6 ± 0.5) (ANOVA *p* = 0.694). Vascular injury scores were also comparable at 24 weeks (2.4 ± 1.1 vs. 1.7 ± 0.8 vs. 2.4 ± 0.9) (ANOVA *p* = 0.165).

**Figure 11:**
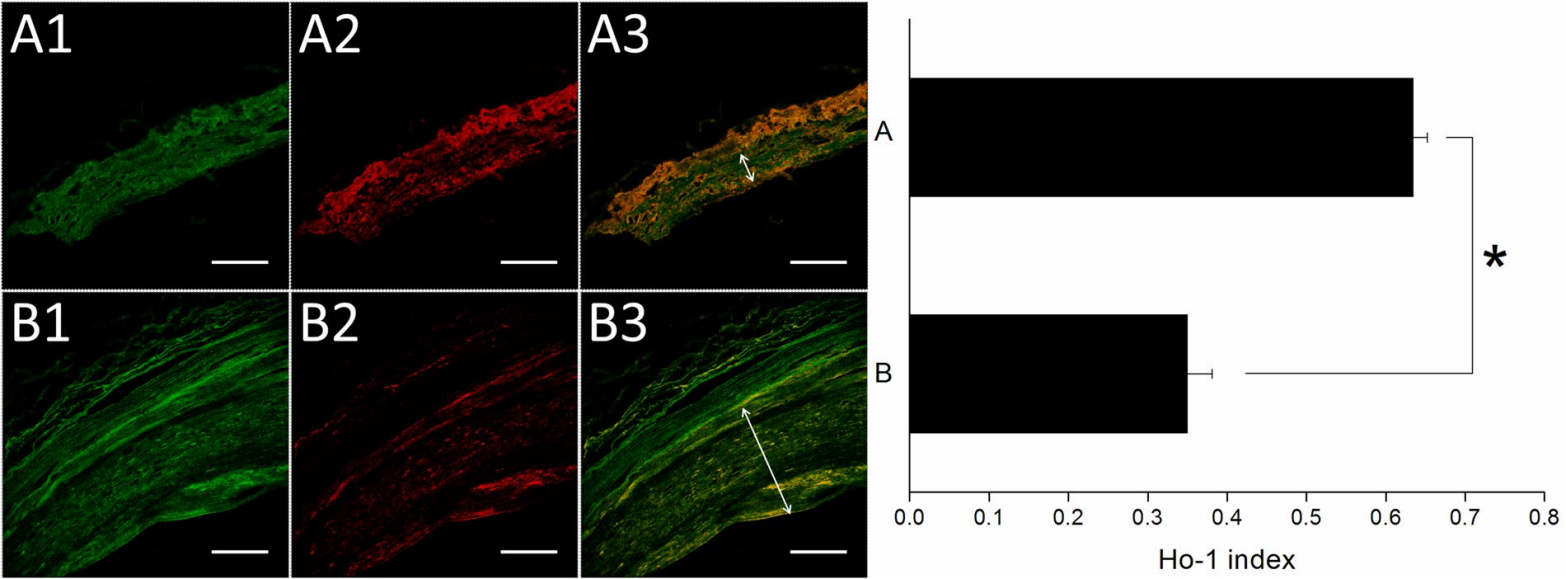
Immunofluorescence of Ho-1 on stented arteries following 24 weeks. Ho-1 immunostaining (red) of group A (**A**) and group B (**B**) stented region and autofluorescence on tunica media (green) were also shown. Great expression of positive labeling with Ho-1 was detected on the rapamycin-eluting cable tie stented vessels. The double arrow illustrates tunica media. Group B showed that substantial neointimal hyperplasia (Scale bar = 150 *μ*m).

**Figure 12:**
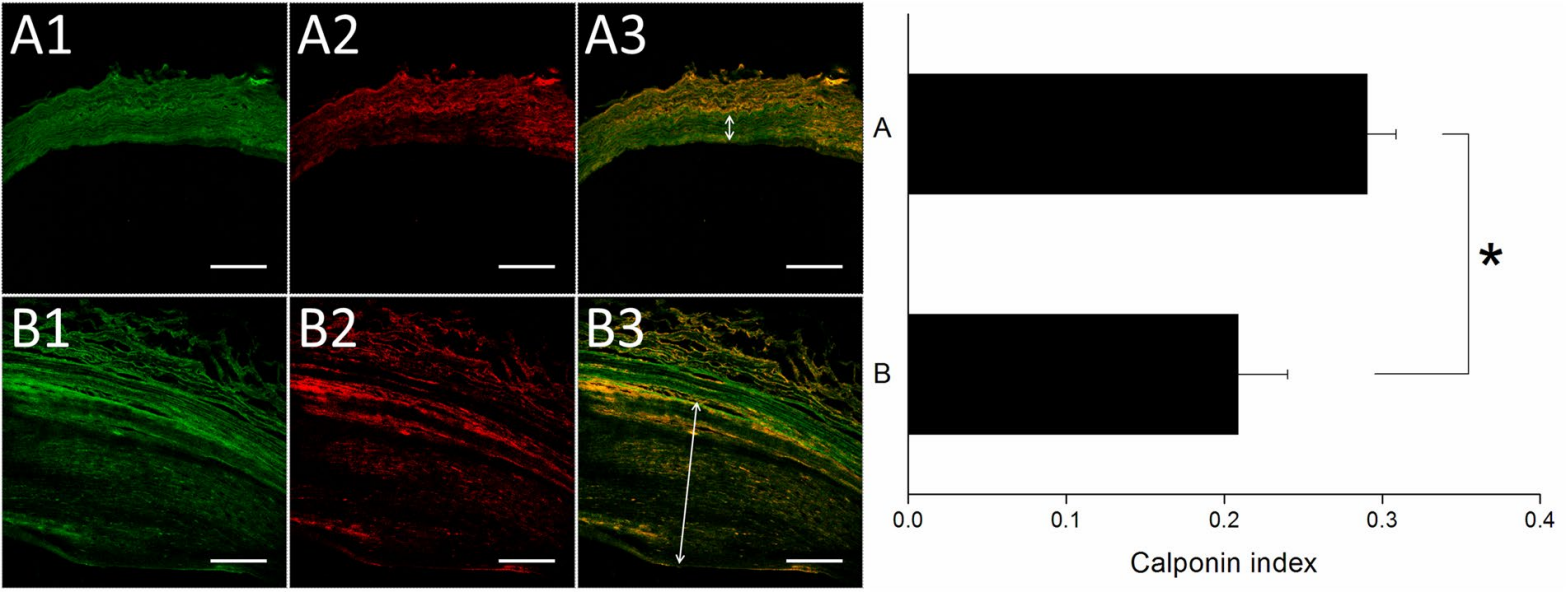
Calponin immunostaining (red) of group A and group B was shown. Less degree of positive labeling with calponin was shown on group B after 24 weeks. The double arrow also depicts tunica media. (Scale bar = 150 *μ*m).

**Figure 13:**
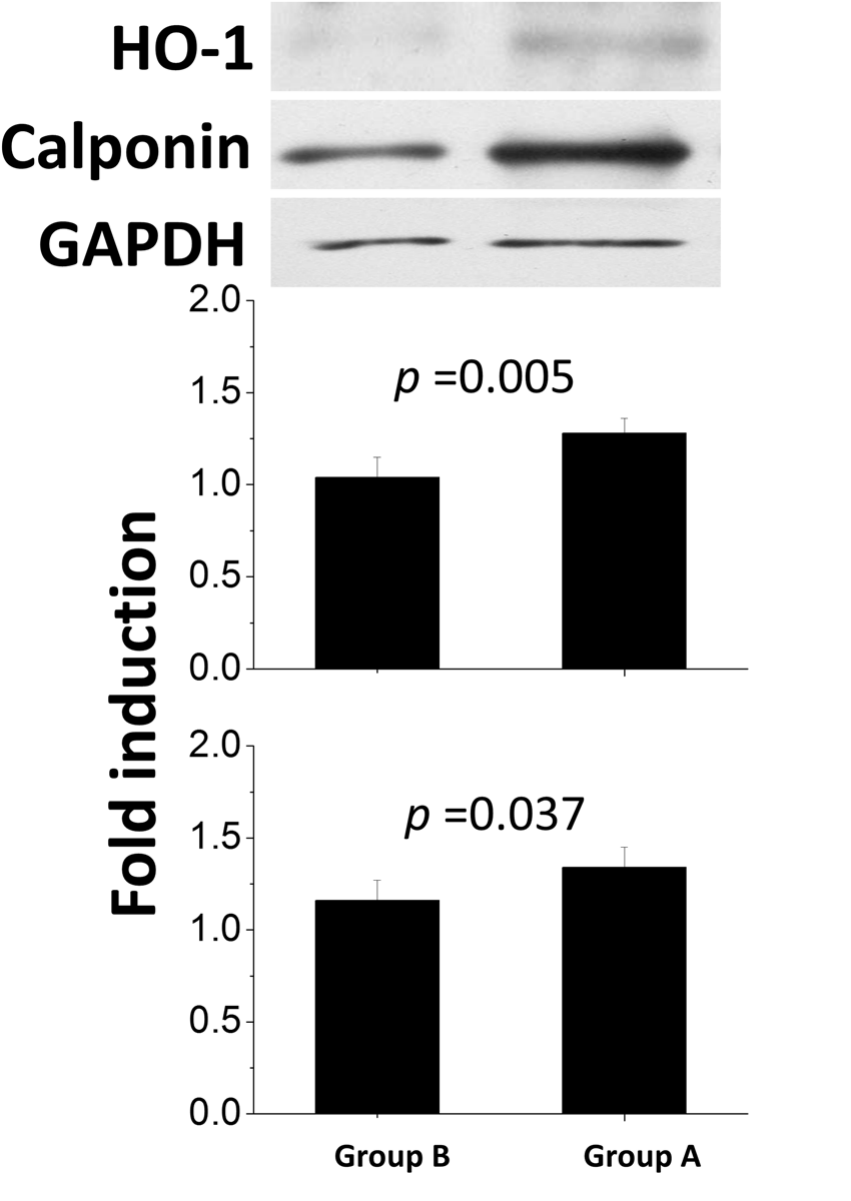
Quantitative analysis of Hol 1 and calponin content of arteries in groups A and B at week 24.

Immunofluorescent labeling of HO-1 and calponin were detected on stented arteries using confocal fluorescence microscopy. HO-1 (Fig. 11) and calponin (Fig. 12) labeling index of the interstitial of intima and elastic lamina of the artery were measured as the ratio of density staining for both markers to DAPI-labeled nuclei. At month six, both groups exhibited significantly higher amount of HO-1 and calponin than did those in the group B (HO-1: group A 0.64 ± 0.02, group B 0.35 ± 0.03; Calponin: group A 0.29 ± 0.02, group B 0.21 ± 0.03) (*p* all < 0.001). In Fig. 13, immunoblotting of level of HO-1 and calponin also was elevated on group A comparing with group B (*p* = 0.005 and *p* = 0.037, respectively).

Six months after the deployment of the fabricated stents, no migration of the implanted stents was observed. In addition, studies using trans-abdominal vascular ultrasound and angiography showed that the stents were all patent in the test rabbits (Supplemental Video).

**Figure 14:**
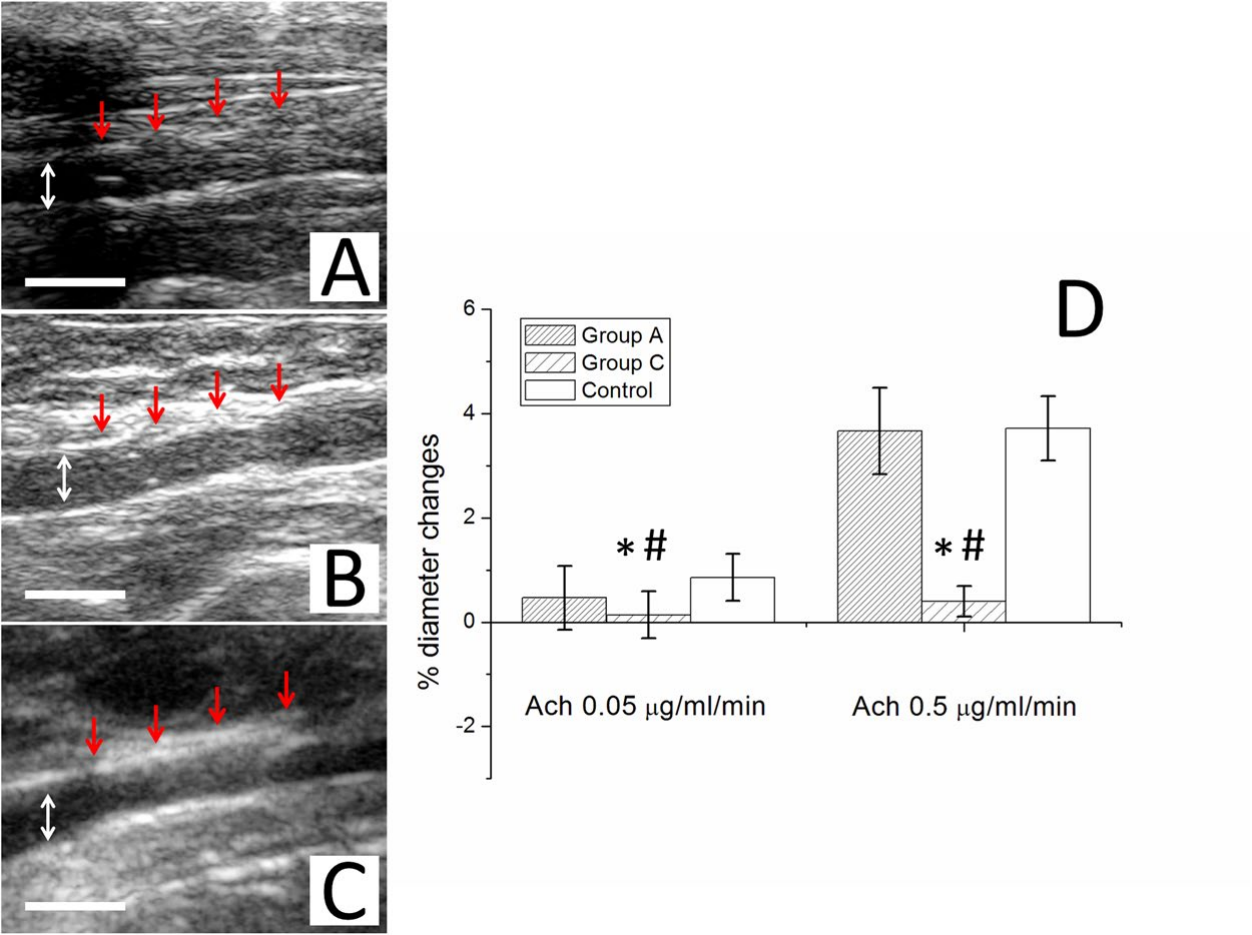
One week (**A**) and 4 weeks (**B**) group A; C: group C) following the procedure, no migration and patent abdominal aorta were observed for the implanted stent (arrow) using the vascular ultrasound. (Scale bar = 5 mm) (*P < 0.05 group A versus group (**C**) in post hoc analysis; ^#^ *P* < 0.05 group C versus control group in post hoc analysis).

Endothelial function was assessed following 28 days of stent deployment, and the response of an endothelia-dependent vasodilatation to Ach was significantly stronger in the rabbits that received cable-tie DES than in those in group C (*post hoc p* < 0.001). The cable-tie rapamycin-eluting biodegradable stents also exhibited a comparable vasodilatory response to the control group (non-injured vessels) (*post hoc p* = 0.241 and 0.902 with Ach 0.05 and 0.5 μg/min/kg infusion, respectively) (Fig. 14).

## Discussion

Routine implantation of balloon-expandable stents to achieve optimal stent dimensions has been reported to improve late clinical and angiographic outcomes^27, 28^. The biodegradable rapamycin-eluting stent that we developed in this study is designed to be expanded by a balloon, and has a self-locking characteristic which can avoid stent recoil resulted from polymer properties and the external pressure of the arteries. The use of oversized balloons and high pressures of inflation can result in late lumen loss and neointimal proliferation. Several studies have confirmed that the main predictors of freedom from restenosis following stent implantation are the minimal luminal area and diameter achieved^29, 30^. In this study, the stent struts become completely expanded and fully apposed at 8 atm, which is comparable to the pressure performed for the nominal pressure at which expands to its named balloon diameter and needed for present stent deployment^10, 31^. Due to the self-locking design, changes in longitudinal dimensions of the fabricated stents following expansion are insignificant. Therefore, the delivery and deployment systems for our proposed stent are using similar deployment techniques and thereby hastening clinical acceptance for endoluminal deployment.

Sufficient radial forces are a crucial element of biodegradable stents, which are anticipated to display minimum radial recoil and high radial force when implanted in various advanced atherosclerotic lesions^32^. Neither the collapse pressure nor compressive strength of our fabricated biodegradable stents reduced significantly after immersion in buffer solution, which demonstrated that these characteristics remained almost unaltered in all the study period. The ultimate collapse pressure needed to oppose arterial blood vessel pressure was 3 × 10^4^ Pa (0.31 kg/cm^2^)^33^, which is lower than the pressure of our PLLA biodegradable stent design (around 1.36 kg/cm^2^). The collapse pressure of our polymeric stent was also markedly higher than the human physiological pressure (1.02 kg/cm^2^)^12^. In addition, no significant reduction in weight was noted in the stents after immersion in PBS for 16 weeks. This may be because the total degradation time of PLLA has been reported to be more than 2 years^34, 35^, which indicates that minimal degradation and water uptake takes place to initiate hydrolysis of PLLA.

Rapamycin is an effective immunosuppressant and retains both antineoplastic and antifungal properties. Rapamycin for local anti-proliferative therapy can inhibit the migration and growth of arterial smooth muscle cell, and prevent intimal hyperplasia from restenosis after balloon angioplasty^36, 37^. The sustained release of rapamycin from our biodegradable stents could significantly decrease smooth muscle cell growth as well as reduce inflammatory reactions *in vivo*, which are the main reasons of stent restenosis and neointimal proliferation.

In this study, PLGA was electrospun into nanofibers to elute rapamycin onto the surface of biodegradable cable-tie stents to deliver drug locally. The fiber diameters reduced with the addition of rapamycin. This might be due to the fact that the polymer concentration in the PLGA solution deceases with the addition of the drugs. The solution thus has less strength to resist the action of the external stretching force by the electrical field during electrospinning. Electrospun fiber diameters decreased accordingly. Many various polymeric ingredients are used because of they can perform a continuous delivery of pharmaceuticals, of which PLGA is the most promising biodegradable bio-material because of non-toxic and minimal inflammatory responses. Venkatraman *et al.* developed drug-eluting biodegradable stents using a solvent-casting method, and reported that the collapse pressure of PLLA stents reduced with a more significant decrease at a higher level of drug combination^38^. Therefore, drug concentrations should be restricted to less than 1 wt% to maintain the mechanical characteristics of the PLLA matrix. The use of electrospun nanofibers has advantage of the increased drug loading of the PLLA stent with no significant decrease in mechanical strength. The experimental results of this study also indicated that the biodegradable stents could release an effective and high concentration of rapamycin for over 4 weeks. Furthermore, by using electrospun nanofibers, the initial burst delivery of rapamycin amount was small, thereby avoiding the possible toxicity related to rapamycin^39^ and adding benefits in the light of constant drug delivery for vascular uses.

HO-1 is an adaptive enzyme, whose production is triggered in oxidative stress-induced inflammatory^40^. HO-1 has directly effects on the vasoconstriction reduction and cell proliferation inhibition during vascular injury^41^. Vascular wound healing considered as local reparative inflammatory responses implicates an essential protective role for HO-1 in arterial vascular disease. Additionally, rapamycin has therapeutic effect on the induction of HO-1 expression for the antiproliferative actions^42^. Calponin, a vascular smooth muscle cell differentiation marker, is a protein associated with actin, calmodulin, and tropomycin and is involved in the ancillary role of smooth muscle cell contraction^43, 44^. Expression of the calponin is reduced in proliferating smooth muscle cells and resultant atherosclerotic neointima in response to injured arterial media^45, 46^. Hence, rapamycin can promote and induce vascular smooth muscle cell differentiation with increased calponin and is an inhibitor of instent restenosis resulted from vascular smooth muscle cell proliferation^47^.

There are some limits to this study. First, only a relatively small number of experimental animals were included, and we used denuded rabbit aortas without pre-existing atherosclerotic lesions. Second, the follow-up period in our study was 6 months after cable-tie stent implantation, and thus it remains unknown whether endothelial dysfunction associated with rapamycin release persists beyond 6 months. However, vascular function at the segment of the stent was preserved, thereby confirming endothelial recovery. The recovery of the functionality of the endothelium, as observed at 1 month with complete endothelial coverage at 6 months, may maintain the vasoprotective effect^48, 49^. In addition, the long-term outcomes of the use of our proposed stent are unknown, and further studies with a long follow-up period are warranted.

## Conclusions

We developed a biodegradable “cable-tie” type eluting rapamycin nanofiber stent. The design included a self-locking mechanism and the ability to avoid stent recoil resulted from polymer properties and external pressure of blood vessels. The fabricated cable-tie stents exhibited excellent mechanical properties on evaluation of compression test and collapse pressure, and less than 8% weight loss following being immersed in PBS for 16 weeks. Furthermore, the biodegradable stents delivered high rapamycin concentrations for over 4 weeks and achieved substantial reductions in intimal hyperplasia associated with elevated heme oxygenase-1 and calponin level on the denuded rabbit arteries during 6 months of follow-up. The cable-tie type stents developed in this study might have high potential impacts for the local drug delivery to treat various vascular diseases.

## Electronic supplementary material

Video legend

Supplemental Video
