# Supplementary material for: Biodegradable Cable-Tie Rapamycin-eluting Stents
Source: Sci Rep. 2017 Mar 8;7:111. doi: 10.1038/s41598-017-00131-w (PMC5427919; doi:10.1038/s41598-017-00131-w)
Supplement: Supplementary file 1 — Video legend [file 41598_2017_131_MOESM1_ESM.doc]

**Biodegradable Cable-Tie Rapamycin-eluting Stents**

Cheng-Hung Lee^1^, Ming-Jer Hsieh^1^, Shang-Hung Chang^1^, Chang-Lin Chiang^2^, Ching-Lung Fan^2^, Shih-Jung Liu^2,3*^, Wei-Jan Chen^1^, Chao-Jan Wang^4^, Ming-Yi Hsu^4^, Kuo-Chun Hung^1^, Chung-Chuan Chou^1^, Po-Cheng Chang^1^

^1^Division of Cardiology, Department of Internal Medicine, Chang Gung Memorial Hospital-Linkou, Chang Gung University College of Medicine, Taiwan

^2^Department of Mechanical Engineering, Chang Gung University, Taiwan

^3^Department of Orthopedic Surgery, Chang Gung Memorial Hospital-Linkou, Taiwan

^4^Department of Medical Imaging and Intervention, Chang Gung Memorial Hospital-Linkou, Taiwan

*To whom correspondence and reprint requests should be addressed:

Shih-Jung Liu, Ph.D.

Biomaterials Laboratory, Mechanical Engineering

Chang Gung University

259, Wen-Hwa 1^st^ Road

Kwei-Shan, Tao-Yuan 333

Taiwan

Tel: +886-3-2118166 Fax: +886-3-2118558

Email: shihjung@mail.cgu.edu.tw

**Video legend**

Using trans-abdominal vascular ultrasound for the patency of stents after 6 months.
